# scRDEN: single-cell dynamic gene rank differential expression network and robust trajectory inference

**DOI:** 10.1038/s41598-025-01969-1

**Published:** 2025-05-15

**Authors:** Han Zhang, Wei Zhang, Xiaoying Zheng, Yuanyuan Li

**Affiliations:** School of Mathematics and Physics, Wuhan Institute of Technology, Wuhan, 430073 China

**Keywords:** Single-cell analysis, Gene regulatory network, Trajectory inference, Computational biology and bioinformatics, Systems biology

## Abstract

The remarkable advancement of single-cell RNA sequencing (scRNA-seq) technology has empowered researchers to probe gene expression at the single-cell level with unprecedented precision. To gain a profound understanding of the heterogeneity inherent in cell fate determination, a central challenge lies in the comprehensive analysis of the dynamic regulatory alterations that underlie transcriptional differences and the accurate inference of the differentiation trajectory. Here, we propose the method scRDEN, a robust framework that infers important cell sub-populations and differential expression networks of multiple genes along the differentiation directions of each branch by converting the unstable gene expression values in cells into relatively stable gene-gene interactions (global features) and extracting the order of differential expression (network features), and further integrating the expression features of different dimension reduction methods. When applied to five published scRNA-seq datasets from human and mouse cell differentiation, scRDEN not only successfully captures the stable cell subpopulations with potential marker genes, measures the transcriptional differences of gene pairs to identify the rank differential expression network along the differentiation direction of each branch. In addition, in multiple gene rank differential expression networks, the rank expression directly related to transcription factors/marker genes shows a significant strengthening and weakening trend along with their expression changes, and the distribution of diversity and cluster coefficient show a non-monotonic change trend, including the cases of increasing first and then decreasing or decreasing first and then increasing. This may correspond to the mechanism of cells gradually differentiating into stable functions. It is particularly noteworthy that scRDEN method yielded exceptional results when applied to the large-scale, multi-branched, double-batch mouse dentate gyrus data. This outstanding performance provides novel and valuable insights into large-scale, multi-batch trajectory inference and the study of transcriptional mechanism regulation during the processes of differentiation and development.

## Introduction

The rapid development of single-cell transcriptome sequencing technology allows researchers to study cell state transitions and various biological processes at single-cell resolution^1^ and opens new fields for exploring the basic mechanisms of normal development and disease^2,3^. It further allows researchers to measure gene expression levels within individual cells, identify specific cell types within complex cellular populations, analyze heterogeneity in single cells, and describe gene expression trends during cellular differentiation^4,5^. This provides new insights into cell differentiation, development, disease, and other complex cellular processes^6^. Several computational methods pertaining to dimension reduction, clustering, and pseudo-time reconstruction have been developed for the analysis of single-cell (sc) RNA sequencing data. Based on these methods, it is possible to discover new or rare cell types in complex tissues and study cell differentiation processes^6–9^, most of these methods primarily focus on the analysis of gene expression levels. However, many biological processes involve the interactions between genes. Studying the expression networks among genes and cell differentiation lineages will greatly contribute to revealing the heterogeneity of cells and the mechanism of cell fate decision^10^.

Inferring gene regulatory networks (GRNs) is one of the key issues in studying single-cell transcriptome data and has developed several methods^11^. Constructing gene regulatory networks can help identify key factors that influence biological fate, such as factors that control cell differentiation and determine phenotypes in disease progression. Identifying the relationships between genes through gene regulatory networks can help understand functional heterogeneity in different cell populations and pinpoint the key genes that drive heterogeneous cell functions^12,13^. The correlation network of scRNA-seq data can measure gene-gene associations based on correlation coefficients and is suitable for large and high-dimensional datasets^11^. Li^14^ uses the Pearson coefficient to derive the correlation between mRNA and lncRNA to construct a co-expression network to study the pathogenesis of tumors. Ranjan^15^ employs Pearson’s coefficient to measure the correlation between genes for the purpose of screening highly variable genes. Yang^16^ reveals the common system-level properties of prognostic genes for different cancer types from the gene co-expression network. There are also some methods to construct dynamic networks to analyze the dynamic changes in biological processes. Matsumoto et al.^17^ present a highly efficient optimization algorithm to reconstruct expression dynamics and infer GRNs from differentiating cells. Different from other approaches, Dai^10^ proposes a cell-specific network for each cell by considering statistical independence. Zhong^18^ develops scGET by utilizing the cell-specific network to detect the signal of a critical transition or cell fate commitment during the embryonic differentiation process and identify non-differentially expressed genes. Other methods such as Ye^19^ develops the convolutional neural network based on gene co-expression for better inference of gene relationships. Huynh-Thu et al.^20^ develop a tree-based algorithm GENIE3, which adopted a distinctive way to infer regulatory networks. The ESCO simulator adopts the concept of copula to impose gene co-expression^21^. The performance of some mature methods in high-noise and high-dimensional datasets remains to be confirmed^12–17^. For example, the Pearson coefficient only measures the linear association between genes and cannot fully distinguish the causal relationships between genes. Although cell-specific networks can be used by existing single-cell methods^18,19^ to identify important transitions during the differentiation process, the accuracy and stability of the results may be affected by the exact timing of these changes and the high cell heterogeneity. To effectively infer the interaction relationships between genes, the information on the differentiation order of cell populations and the differential changes in the gene-pair structure during differentiation also need to be considered.

Pseudo-time trajectory inference, a crucial task in single-cell data analysis, can arrange the sequence of individual cells based on single-cell data and enables the description of temporal trends in the gradual transition of single cells by ordering them along the developmental trajectory^4^. In recent years, researchers have invented a variety of pseudo-time trajectory inference algorithms, while developing a unified framework to benchmark pseudo-time trajectory inference algorithms^22^. TSCAN constructs the minimum spanning tree (MST) based on the center of mass of cell clusters and then infers the pseudo-time ordering of the cells, which reduces the complexity of the tree space^23^. Originally used to reconstruct the differentiation trajectories of human skeletal muscle myoblasts, Monocle has a limitation in that it cannot predict bifurcations in the pedigree, thus being able to analyze only linear differentiation systems^24^. This problem has been addressed in the improved Monocle2, one of the most dominant pseudo-time inference trajectory algorithms available, which is based on the DDRTree algorithm iteratively determining the stable position of the cell projections in a low-dimensional space and generating MST to represent the inferred lineage structure^25^. SLICER begins by constructing a k-nearest neighbors graph between cells and then identifies the shortest path between the initial and final cells as the pseudotemporal trajectory^26^. Recently, researchers have proposed a likelihood-based kinetic model, scVelo, that can extend RNA velocity to non-steady-state systems with different kinetics. Furthermore, it defines shared latent times for genes representing an internal clock within cells^27^. However, these single-cell pseudo-time trajectory inference methods have significant drawbacks. Most of these methods heavily rely on the accurate identification of cell populations. Moreover, these methods lack robustness and accuracy. They are highly sensitive to data noise, which can distort the distance-based calculations employed in many of these algorithms, and they face difficulties in handling complex biological processes like multi-level branching and overlapping differentiation paths.

In this study, we developed scRDEN (Single-cell gene rank differential expression network) to overcome the limitations of existing methods. Unlike most existing approaches that solely depend on cell-based features, scRDEN constructs a more stable network by leveraging gene-gene correlations. Specifically, by concentrating on gene-pair correlations, scRDEN is less susceptible to cell population misidentification, which allows it to more effectively capture the underlying biological regulatory relationships. The gene-based network construction of scRDEN not only enhances its stability by mitigating the impact of data noise but also enables it to better handle multi-branch complex biological processes. This is achieved by identifying key genes and their regulatory relationships that drive different differentiation branches. As a result, scRDEN can be employed to identify cell subpopulations and infer the gene rank differential expression network of different differentiation trajectories in more complex differentiation processes. We can evaluate scRDEN using five real datasets and compare its performance with that of existing methods in terms of robustness and accuracy, thereby providing a more reliable and powerful tool for single-cell data analysis.

## Results

**Fig. 1:**
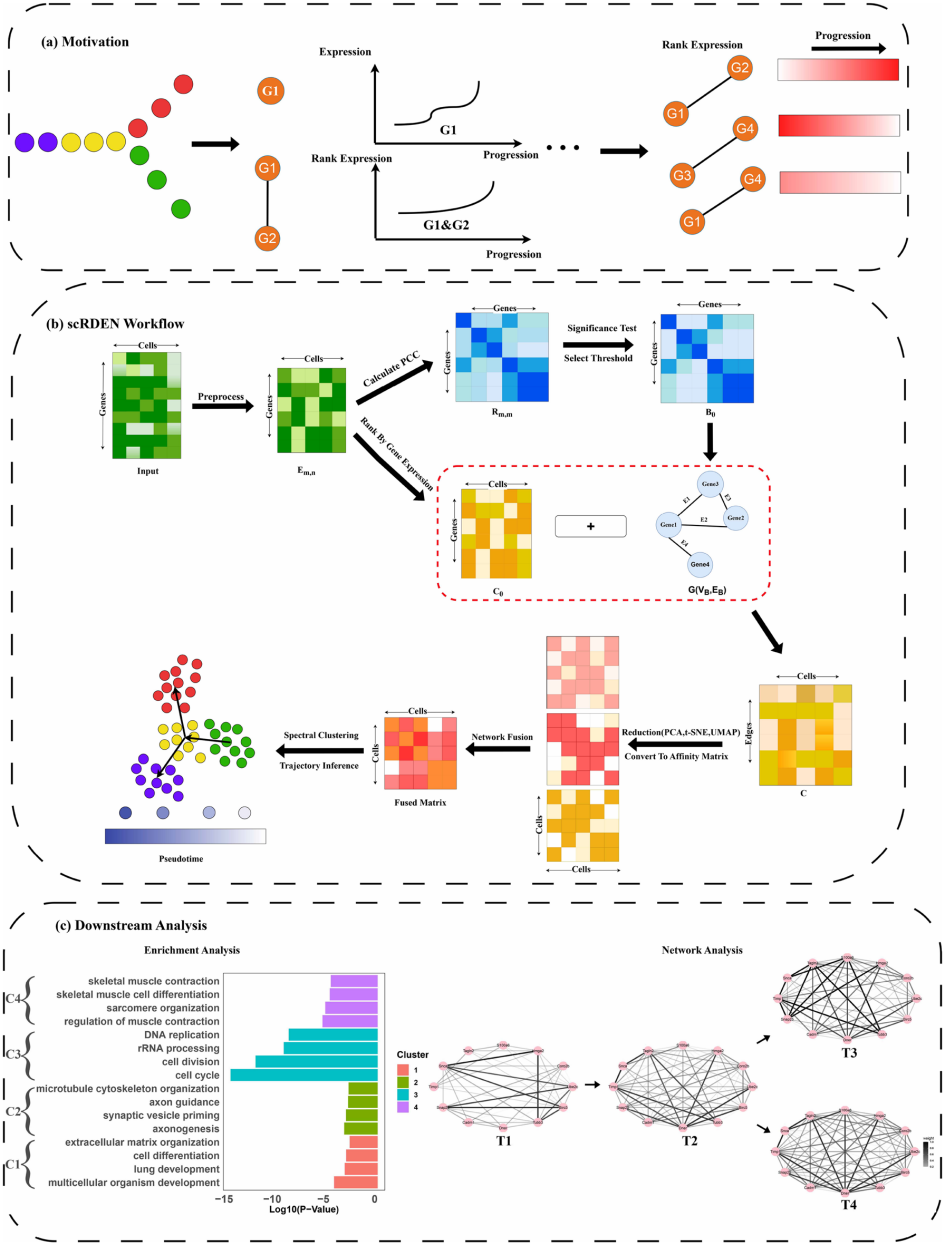
The overall analysis of scRDEN encompasses three parts. (**a**) Motivation. Compared with the differential expression of individual genes in single-cell data, the differential expression of gene pairs is more stable and has stronger robustness during the cell differentiation process. (**b**) scRDEN Workflow. It demonstrates the entire process starting from the single-cell transcription matrix and ultimately achieving clustering analysis and trajectory inference. (**c**) Downstream Analysis. It includes the enrichment analysis of differentially expressed genes and the dynamic gene rank differential expression network identification along with the different directions of differentiation.

We developed scRDEN method to robustly identify cell subpopulations and infer pseudo-time trajectory based on stable relative expression ordering^28^. The overall framework of scRDEN is depicted in Fig. 1. As demonstrated in Fig. 1a, the expression levels of individual genes exhibit unstable variation across different cells. In contrast, the expression order of reliable gene pairs shows greater robustness. As shown in Fig. 1b (see “Methods”), the scRDEN workflow involves constructing a gene rank differential expression network using a rank-sorted matrix and a gene co-expression network, subsequently identifying cell subpopulations and assigning pseudo-time to each cluster. Furthermore, our method facilitates functional characterization of genes associated with critical biological processes. As illustrated in Fig. 1c, we performed enrichment analysis and dynamic rank differential network analysis for downstream investigations.

To demonstrate the effectiveness of scRDEN, we applied it to five real scRNA-seq datasets from Mus musculus and Human. The first dataset consists of 101 mouse lung alveolar type 2 (AT2) cells with cell type assignments of ‘AT2’, ‘Sftpc+’ or ‘Sftpc+Scgb3a2+’ selected at four developmental time points (E14.5, E16.5, E18.5, adult)^29^. The second dataset is dendritic cell (DC) progenitors datasets, including 57 Monocyte and Dendritic cell Progenitors (MDPs), 89 Common Dendritic cell Progenitors (CDPs), and 92 Pre-Dendritic Cells (PreDCs)^30^. The third dataset is fibroblast-reprogramming-treutlein (Fibroblast) datasets, containing 355 cells directly reprogrammed from mouse embryonic fibroblasts (MEF) to induced neuronal (iN) cells on days 0, 2, 5, 20, and 22^31^. The fourth dataset is the germline-human-female weeks datasets (Germline), which contains 666 cells, collected from 4-26 weeks^32^. The fifth dataset is the mouse dentate gyrus dataset, which selected a total of 11926 cells at P0, P5, and P18^33^. The detailed information about the quantification and cell types of these datasets is described in the related articles. In the following, we respectively refer to these five datasets as AT2, DC, Fibroblast, Germline, and Dentate gyrus. In the subsequent analysis, we mainly focus on Fibroblast, Germline, and Dentate gyrus datasets. The details of five datasets are summarized in Table 1, Trajectory types are named from Saelens^22^.

**Table 1:** Description of the single-cell datasets used in data analysis.

Dataset	Number of cell types	Number of cells	Number of genes	Trajectory types	Source
AT2	4	101	23363	Linear	GSE52583
DC	3	238	4480	Linear	GSE60783
Germline	4	666	5319	Linear	GSE86146
Fibroblast	7	355	3379	Bifurcation	GSE67310
Dentate gyrus	24	11926	13999	Multifurcation	GSE104323

The table shows the number of cell types, number of cells, number of genes, trajectory types and source.

### scRDEN robustly inferred three subgroups, pseudo-time, and dynamic regulatory networks of female fetal germ cells differentiation processes

**Fig. 2:**
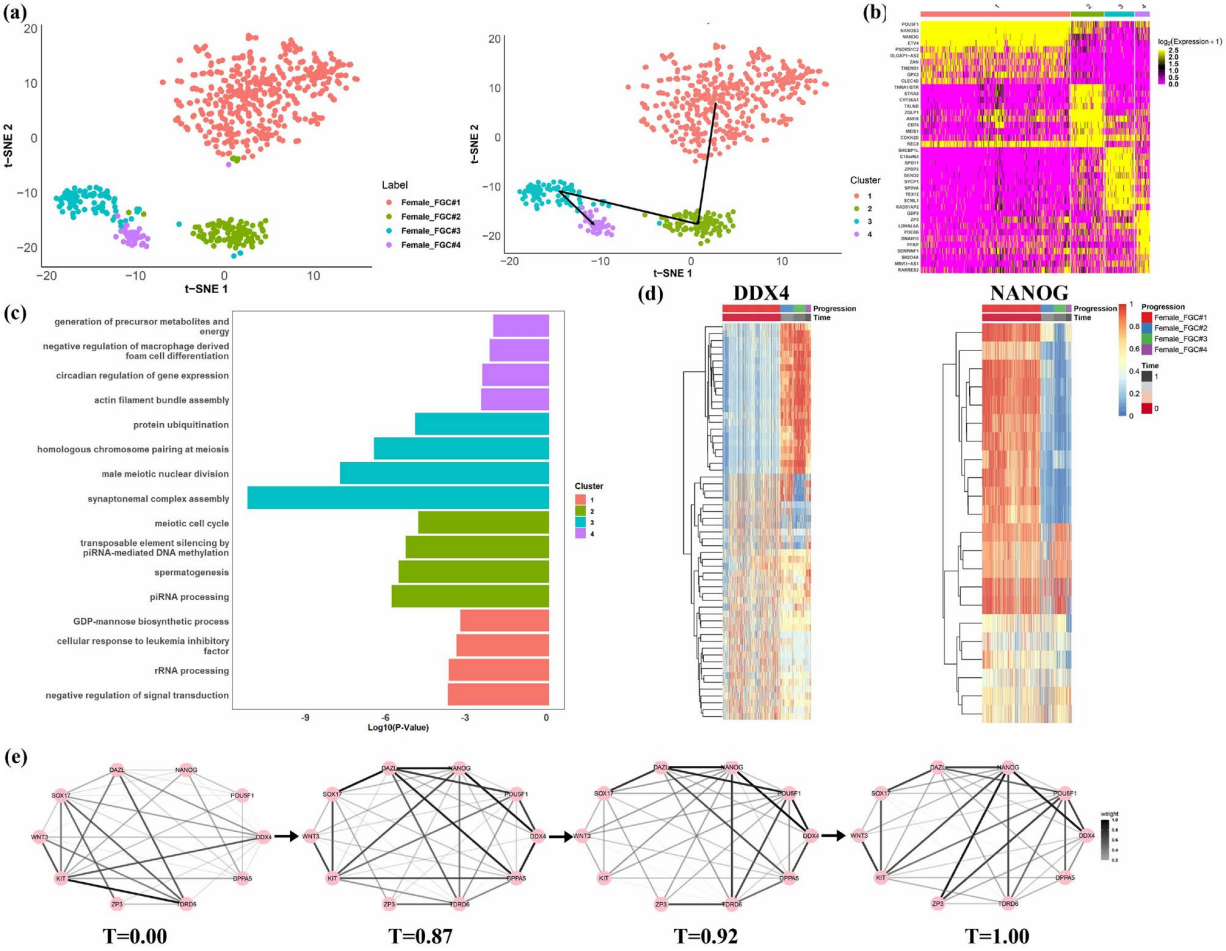
During the process of female fetal germ cell differentiation^32^, scRDEN identifies four cell populations, and related marker genes and infers the four differential expression networks along the unidirectional differentiation trajectory. (**a**) Cells are visualized on the first two t-SNE components and colored by developmental stages and identified clusters with an order (C1$\rightarrow$C2$\rightarrow$C3$\rightarrow$C4) of differentiation noted by the three black arrows. (**b**, **c**) The heatmap of the expression of the top 10 marker genes in each of the four clusters and the key functional annotations of the enriched genes during the differentiation of female fetal germ cells. (**d**) Two heatmaps of gene expression strongly correlated with the NANOG gene and the DDX4 gene, respectively, plotted along cell differentiation and the inferred pseudo-time, have been normalized to the range of 0 to 1 after Minmax standardization. (**e**) Four dynamic rank differential expression networks composed of 10 key genes along the differentiation trajectory of female fetal germ cells from the initial stage (T = 0.00) to the terminal stage (T = 1.00), where a larger mean value of the normalized rank corresponds to a thicker edge.

As the first demonstration, scRDEN were applied to study the development of female fetal germ cells(FGC)^32^, which consists of 666 individual cells with lineage of unidirectional differentiation from four developmental stages: FGC${\#1}$(Mitotic), FGC${\#2}$(RA responsive), FGC${\#3}$(Meiotic), FGC${\#4}$(Oogenesis). scRDEN classifies most of Female${\_}$FGC${\#2}$ are combined into C1 (434 cells), C2 consists of Female${\_}$FGC${\#2}$(94 cells) and Female${\_}$FGC${\#3}$ (4 cells), C3 consists of Female${\_}$FGC${\#3}$ (85 cells) and Female_FGC${\#2}$(3 cells), C4 consists of Female${\_}$FGC${\#4}$ (39 cells) and Female${\_}$FGC${\#3}$ (4 cells), further reveal the unidirectional differentiation trajectory in t-SNE reduction space. The initial time point of the C1 is determined by POU5F1 and NANOG gene significant expression, as shown in Fig. S1a. As shown in Fig. 2a, based on the determination of the initial differentiation stage and the trajectory inference method, the four cell clusters with the order of C1$\rightarrow$C2$\rightarrow$C3$\rightarrow$C4 in the t-SNE reduction space display a unidirectional developmental trajectory, which is consistent with the conclusion of the unbranched real trajectory differentiation process in Li^32^. To further elaborate on whether these cell subpopulations are likely to be functional, we identify the significant feature genes associated with each cell subpopulation. We performed the 2809 marker genes (log2FC>1, adjusted p-value<0.05) in total and showed the heatmap of the top 10 marker gene expressions in each subpopulation, which reveals a clear specific-expression pattern in each cluster (Fig. 2b). Then we conduct gene function analysis to identify biological processes significantly enriched by the marker genes from each cluster. As shown in Fig. 2c, DAVID gene ontology analysis^34^ shows that 663 genes in C1 are involved in negative regulation of signal transduction (p-value = 1.55E−04), rRNA processing (p-value = 1.69E−04), cellular response to leukemia inhibitory factor (p-value = 3.31E−04), GDP-mannose biosynthetic process (p-value = 4.57E−04), and 433 genes in C2 are involved in piRNA processing (p-value = 1.18E−06), spermatogenesis (p-value = 2.17E−06), transposable element silencing by piRNA-mediated DNA methylation (p-value = 4.1E−06), meiotic cell cycle (p-value = 1.17E−05)), 1078 genes in C3 are related to synaptonemal complex assembly (p-value = 4.55E−12), male meiotic nuclear division (p-value = 1.36E−08), homologous chromosome pairing at meiosis (p-value = 2.60E−07), protein ubiquitination (p-value = 8.97E−06). Similarly, the functional enrichment of 6634 important genes in C4 is associated with actin filament bundle assembly (p-value = 2.72E−03), circadian regulation of gene expression (p-value = 3.10E−03), negative regulation of macrophage-derived foam cell differentiation (p-value = 5.79E−03), generation of precursor metabolites and energy (p-value = 7.92E−03). This pattern indicates that the developmental process involves dynamic reorganization of the cytoskeleton and requires substantial energy and metabolic support when FGCs start oocyte formation and folliculogenesis^35^.

Pathway enrichment analysis showed that the marker gene NANOG is enriched in somatic stem cell population maintenance and the marker gene DDX4 is enriched in piRNA processing. To probe the possible regulatory relationship of the NANOG (transcription factor) and DDX4 (RNA helicase) genes in these pathways and to better explore the biological significance of these genes, we select some genes with significant rank differential expression from the NANOG and DDX4 genes respectively and show the expression heatmap of them during the unidirectional differentiation process (Fig. 2d). We observe that 22 connections associated with the NANOG gene, such as NANOG&ESCO2, is initially highly expressed at the initial stage (T = 0.00), and then rapidly decrease from the early post-differentiation stage (T = 0.87) to the terminal stage of differentiation (T = 1.00). The 58 connections associated with the DDX4 gene have opposite expression patterns, with low expression at T = 0.00, then increase from pseudo-time 0.87 to 1. Interestingly, we further find that the expression values of these genes with significant differential expression either increase or decrease along the inferred pseudo-time direction, which may imply significant changes in the gene differential expression patterns. The unidirectional differentiation trajectory of female fetal germ cell: C1$\rightarrow$C2$\rightarrow$C3$\rightarrow$C4 could be used to explore the dynamic changes in network topology of differential expression at different time points (Fig. 2e). The key 10 genes (POU5F1, NANOG, DDX4, DAZL, SOX17, KIT, WNT3, ZP3, TDRD6, DPPA5) have been chosen to measure the changes in regulatory relationships when they play important roles in expression regulation and have highly differential expression with transcription factors during the FGC proliferation^32^. As shown in Fig. 2e, we can observe and utilize the diversity coefficient and the clustering coefficient to measure the significant changes that occur in the differential expression network among genes along different differentiation stages. Late FGC marker genes, such as DDX4, DAZL, and TDRD6, are expressed in the RA-responsive phase, meiotic prophase, and oogenesis phase FGCs^36^, consistent with the enhanced linkage strength of these genes with obvious differential expression in the network from T=0.87 to 0.92 and then to 1.00. In Figs. S1b and S1c, the diversity and cluster coefficient of gene expression differential networks show a tendency to increase and then decrease during the pseudo-time course of FGC, which is consistent with the conclusion that female fetal germ cells have a higher potential for differentiation with more intense biological activity in the early stages of development and then cellular activity decreases and tends to stabilize in the later stages of development^32^. The result demonstrates that scRDEN is capable of inferring important cell clusters, and the gene-gene differential expression network containing important transcriptional relationships along the real trajectory from female fetal germ cell differentiation.

### scRDEN robustly inferred three subgroups, pseudo-time, and dynamic regulatory networks of mouse embryonic fibroblasts differentiation processes

**Fig. 3:**
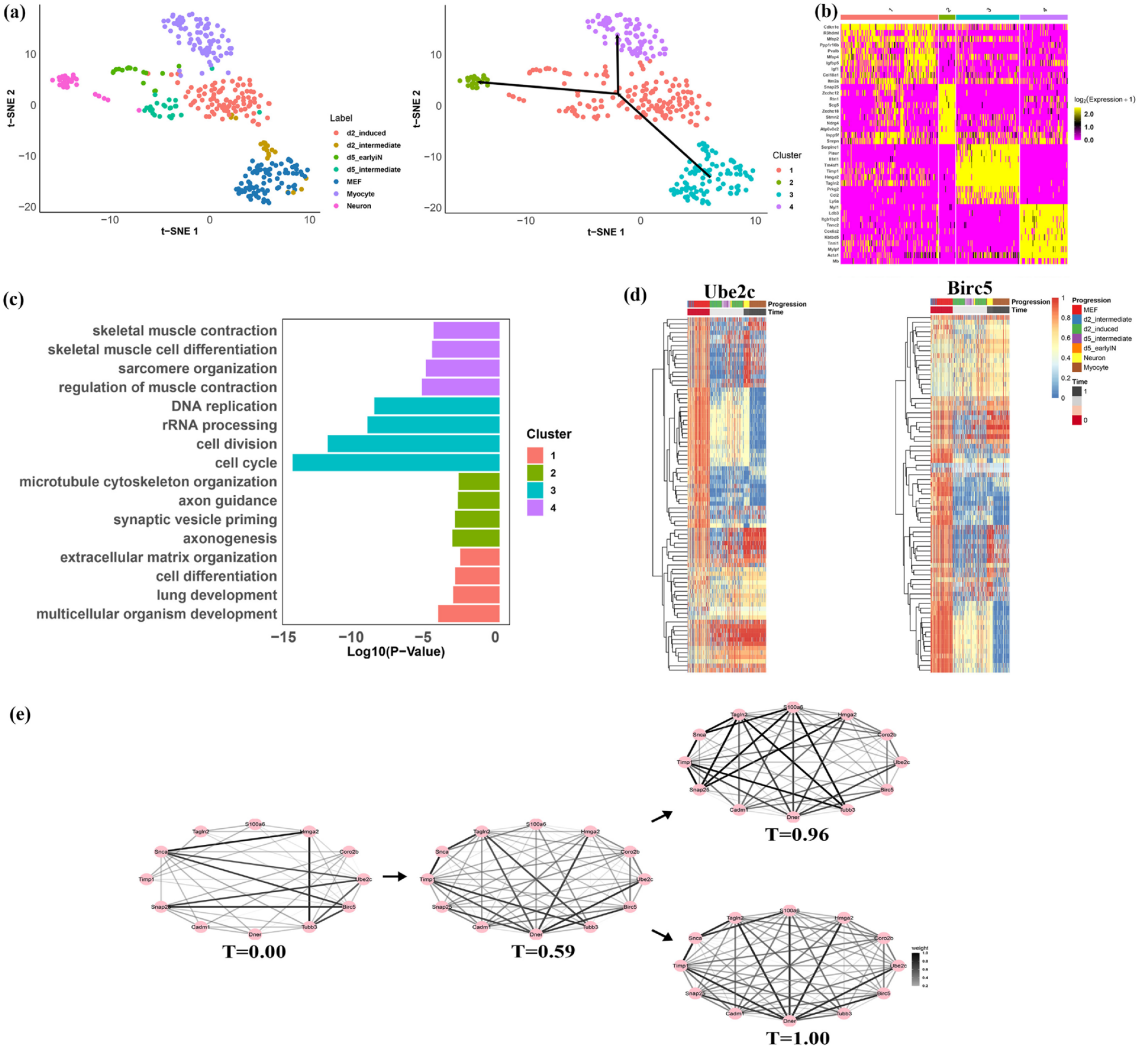
During the process of mouse embryonic fibroblasts development^31^, scRDEN identifies four cell populations and related marker genes and infers the four differential expression networks along the double-branched differentiation trajectory. (**a**) Cells are visualized on the first two t-SNE components and colored by developmental stages and identified clusters with two directions of differentiation (C3$\rightarrow$C1$\rightarrow$C2 and C3$\rightarrow$C1$\rightarrow$C4) noted by the three black arrows. (**b**, **c**) The heatmap of the expression of the top 10 marker genes in each of the four clusters and the key functional annotations of the enriched genes during the differentiation of mouse embryonic fibroblasts development. (**d**) Two heatmaps of gene expression strongly correlated with the Ube2c and the Birc5 gene, respectively, plotted along cell differentiation and the inferred pseudo-time, have been normalized to the range of 0 to 1 after Minmax standardization. (**e**) Dynamic rank differential expression networks of the 10 key genes along the differentiation trajectory, where the larger mean value of normalized rank leads to a thicker edge. Four dynamic rank differential expression networks composed of 10 key genes along the two differentiation directions from the initial stage (T = 0.00) to the two terminal stages (T = 0.96 and T = 1.00), where a larger mean value of the normalized rank corresponds to a thicker edge.

As the second demonstration, we further applied scRDEN to analyze the mouse embryonic fibroblasts development^31^, which consists of 355 individual cells from seven developmental stages: mouse embryonic fibroblast (MEF), d2${\_}$intermediate, d2${\_}$induced, d5${\_}$intermediate, d5${\_}$earlyIN, Myocyte, and Neuron. By the ensemble clustering method, four cell clusters and two directions of cell differentiation by scRDEN were revealed in t-SNE reduction space, as shown in Fig. 3a. Most of the C1 consists of d2${\_}$induced (98 cells) and d5${\_}$intermediate (24 cells); The C2 consists of Neuron (26 cells); The C3 consists of d2${\_}$intermediate (17 cells) and MEF (83 cells), and the C4 is composed of Myocyte (75 cells) (Fig. 3a). The initial time point of the C3 is determined by Ube2c and Birc5 gene significant expression, as shown in Fig. S2a. As shown in Fig. 3a (C3$\rightarrow$C1$\rightarrow$C4, C3$\rightarrow$C1$\rightarrow$C2), based on the determination of the initial differentiation stage and expression of Snap25 (marker gene for Neuron), Tnnc2 (marker gene for Myocyte) in Figs. S2b and S2c, the four cell clusters in the t-SNE reduction space display a developmental trajectory with two branches, which is consistent with the conclusion of the real trajectory differentiation process in scTIM^37^. Similarly, we identified 994 marker genes (log2FC>1, adjusted p-value<0.05) and made the heatmap of the top 10 marker gene in each cluster to show their expression in different clusters (Fig. 3b). Except for the marker genes in C2, which are partially highly expressed in C1, the marker genes of the other clusters are highly expressed only in the corresponding cluster. As shown in Fig. 3c,DAVID gene ontology analysis^34^ shows that 101 genes in C1 are involved in multicellular organism development (p-value = 2.98E–05), lung development (p-value = 3.76E–04), cell differentiation (p-value = 5.32E–04), extracellular matrix organization (p-value = 1.23E-03), and 195 genes in C2 are involved in axonogenesis (p-value = 3.36E–04), synaptic vesicle priming (p-value = 5.13E–04), axon guidance (p-value = 8.4E–04), micro tubule cytoskeleton organization (p-value = 9.65E–04)), 576 genes in C3 are related to cell cycle (p-value = 5.36E–16), cell division (p-value = 2.08E–13), rRNA processing (p-value = 1.77E–10), DNA replication (p-value =5.8E–10). Similarly, the functional enrichment of 122 important genes in C4 is associated with regulation of muscle contraction (p value = 1.85E–06), sarcomere organization (p-value = 3.63E–06), skeletal muscle cell differentiation (p-value = 1.06E–05), skeletal muscle contraction (p-value = 1.37E–05).

Pathway enrichment analysis showed that the marker gene Birc5 was enriched in cell cycle, cell division, mitotic cytokinesis, and other pathways, the marker gene Ube2c was also enriched in cell cycle, cell division, and regulation of mitotic 9/22 metaphase/anaphase transition. To investigate the differential expression patterns of the Birc5 and Ube2c genes within these pathways and to further elucidate their biological significance, we selected the connections associated with the Birc5 gene and the Ube2c gene. We drew the expression heatmap of the corresponding genes along the differentiation process in Fig. 3d. We observe that the 75 connections associated with the Birc5 gene and the 81 connections associated with the Ube2c gene have similar differentially expressed modules. For example, the expression of the Birc5 &Tnnt1 module is initially high at the initial stage (T = 0.00), and then it rapidly decreases from the middle differentiation stage (T = 0.59) to the two terminal stages of differentiation (T = 0.98 and T = 1.00). Since MEFs exit the cell cycle upon Ascl1 induction, genes (Birc5, Ube2c) involved in mitosis are downregulated or turned off^31^. This demonstrates that the expression values of significantly differentially expressed genes change identically along the inferred pseudo-time in the same cluster of cells. The trajectories of two paths: C3$\rightarrow$C1$\rightarrow$C4 and C3$\rightarrow$C1$\rightarrow$C2 were used to explore the dynamic changes in network topology at the fate decisions along two directions (Fig. 3e). The 12 genes (Ube2c, Coro2b, Hmga2, S100a6, Tagln2, Snca, Timp1, Snap25, Cadm1, Dner, Tubb3, Birc5) are associated with cell division, synaptic maturation, and positive regulation of Fibroblast proliferation and epithelial cells^31,38^. The first branch trajectory, C3$\rightarrow$C1$\rightarrow$C2, corresponds to the differentiation from MEF into Neuron cells; the second trajectory, C3$\rightarrow$C1$\rightarrow$C4, corresponds to the differentiation from MEF into Myocyte cells. As shown in Figs. 3e, S2d, and S2e, both diversity and cluster coefficient of gene expression differential networks exhibit the same trend on two different branches. We can find that the strength of linkage of the marker gene Snap25 to the other genes roughly shows a gradual increase on T = 0, 0.59, 0.96 and reaches an extreme value at T = 0.96, which could indirectly illustrate the high expression of Snap25 in Neuron cells. This result shows that scRDEN could infer the significant cell subpopulation and critical gene-gene expressional network along the complex trajectory with two branches during the mouse embryonic fibroblast development.

### scRDEN robustly inferred four subgroups, pseudo-time, and dynamic regulatory networks of mouse dentate gyrus differentiation processes

**Fig. 4:**
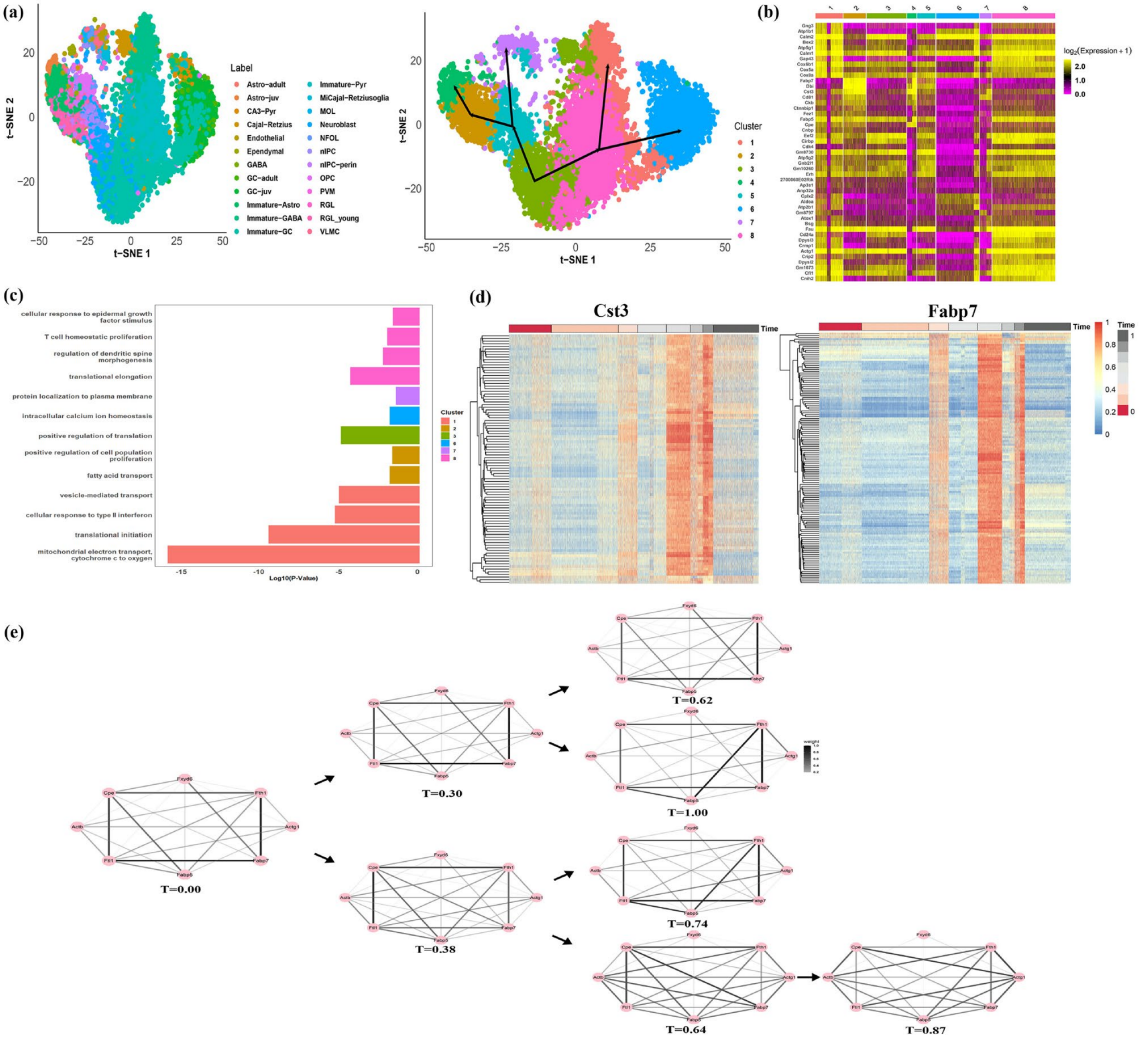
During the process of mouse dentate gyrus development^33^, scRDEN identifies eight cell populations and related marker genes and infers the multi-branching differentiation trajectory and the corresponding gene expression differential networks. (**a**) Cells are visualized on the first two t-SNE components and colored by developmental stages and identified clusters with the four fate decisions (C3$\rightarrow$C5$\rightarrow$C2$\rightarrow$C4, C3$\rightarrow$C5$\rightarrow$C7, C3$\rightarrow$C8$\rightarrow$C1 and C3$\rightarrow$C8$\rightarrow$C6) of differentiation noted by the seven black arrows. (**b**) Heatmap of top 10 marker gene expression each of the eight clusters during mouse dentate gyrus development. (**c**) Key functional annotation for enriched genes in the eight clusters. (**d**) Two heatmaps of gene expression strongly correlated with the Cst3 gene and the Fabp7 gene, respectively, plotted along cell differentiation and the inferred pseudo-time, have been normalized to the range of 0 to 1 after Minmax standardization (**e**) Four dynamic rank differential expression networks composed of 8 key genes along the four fate decisions of dentate gyrus development from the initial stage (T = 0.00) to the terminal stage (T = 1.00), where a larger mean value of the normalized rank corresponds to a thicker edge.

As the last demonstration in large-scale complex multi-branch scRNA-seq differentiation, we further applied scRDEN to analyze the mouse dentate gyrus dataset with two batches (X10X83 and X10X84)^33^, which selected a total of 11926 cells at P0, P5, and P18 with 24 clusters of cells in Fig. S4. In Figs. S4a and S4b, it shows the PCA dimensionality reduction plot without removing the batch effects and the distribution of embedding values of the cells. Figs. S4c and S4d show the dimensionality reduction plot and the distribution of embedding values of the cells after the Harmony^39^ method removes the batch effects. To avoid the batch effects, the Harmony^39^ method was chosen to remove batch effects. We can observe that after the Harmony method eliminates the batch effects, there is less variation among cells from different batches, and the distribution of embedding values for these cells becomes more similar in Fig. S4c and S4d. The data after removing the batch effects is used for the ensemble clustering method, eight cell clusters and four developmental trajectories were identified by scRDEN in t-SNE reduction space (Fig. 4a). Most of the C1 consists of Immature-GABA (599 cells), Immature-Pyr (427 cells), and Immature-GC (318 cells); most of the C2 consists of RGL${\_}$young (446 cells) and Immature-Astro (413 cells); Most of the C3 consists of Immature-GC (697 cells) and Neuroblast (586 cells); Most of the C4 is composed of Immature-Astro (210 cells). The majority of C5 consists of RGL${\_}$young (106 cells), Neuroblast (237 cells), and OPC (165 cells). The majority of C6 consists of GC-juv (1472 cells). Most of C7 is made up of Endothelial (140 cells) and MOL (111 cells). Most of C8 is made up of Immature-Pyr (2354 cells). The initial time point of the C3 is determined by Eomes and Ccnd2 gene significant expression, as shown in Fig. S3a. As shown in Fig. 3a (C3$\rightarrow$C8$\rightarrow$C1, C3$\rightarrow$C8$\rightarrow$C6, C3$\rightarrow$C5$\rightarrow$C7, C3$\rightarrow$C5$\rightarrow$C2$\rightarrow$C4), based on the determination of the initial differentiation stage and expression of Cst3, Fabp7(marker gene for Astrocyte), C1ql2 (marker gene for Granule), Cldn11, Cspg4 (marker gene for Oligodendrocyte), Fhl2 (marker gene for Pyramidal) in Figs. S3b–e, the eight cell clusters in the t-SNE reduction space display a developmental trajectory with four branches, which is consistent with the conclusion of the real trajectory differentiation process in previously studied^33,40^. Due to the large number of low-expressed genes in the data, we identified 342 marker genes and made the heatmap of the top 10 marker genes in each cluster to show their expression in different clusters (Fig. 4b). As shown in Fig. 4c, DAVID gene ontology analysis^34^ shows that 146 genes in C1 are involved in mitochondrial electron transport, cytochrome c to oxygen (p-value = 5.80E–17), translational initiation (p-value = 1.77E–10), cellular response to type II interferon (p-value = 3.50E–06), vesicle-mediated transport (p-value = 6.51E–06), And 12 genes in C2 are involved in fatty acid transport (p-value = 1.17E–02), positive regulation of cell population proliferation (p-value = 1.72E–02), 3 genes in C3 are related to positive regulation of translation (p-value = 8.44E–06). Similarly, the functional enrichment of 4 important genes in C6 is associated with intracellular calcium ion homeostasis (p-value = 1.17E–02). The functional enrichment of 6 important genes in C7 is associated with protein localization to the plasma membrane (p-value = 2.94E–02). The functional enrichment of 149 important genes in C8 is associated with translational elongation (p-value = 3.29E–05), regulation of dendritic spine morphogenesis (p-value = 4.20E–03), T cell homeostatic proliferation (p-value = 8.04E–03), cellular response to cytochalasin B (p-value = 1.20E–02).

Pathway enrichment analysis showed that the marker gene Fabp7 was enriched in fatty acid transport in C2, and the marker gene Cst3 was also enriched in positive regulation of cell population proliferation in C2. To further explore the expression profiles of the Cst3 and Fabp7 genes within these pathways and to further elucidate their biological significance, we select some genes with significant rank differential expression from the gene Cst3 and Fabp7 respectively and show the expression heatmap of them during the differentiation process in Fig. 4d. Due to the excessive number of true cell populations in the dataset, we do not show the ’progression’ label. We observe that 133 connections associated with the Fabp7 gene and the 94 connections associated with the Cst3 gene have the same expression patterns, such as Fabp7 &Dbi is highly expressed at initial stage (T = 0.00), middle stage (T = 0.38) and terminal stage (T = 0.74) of differentiation in the third branch. It is possible because the Astrocyte cells are too dispersed, resulting in the expression of Astrocyte’s marker genes on multiple clusters. The trajectories of the four different paths: C3$\rightarrow$C8$\rightarrow$C1, C3$\rightarrow$C8$\rightarrow$C6, C3$\rightarrow$C5$\rightarrow$C7, and C3$\rightarrow$C5$\rightarrow$C2$\rightarrow$C4, were used to explore the dynamic changes in network topology at different time points (Fig. 4e). The 8 genes (Fxyd6, Fth1, Actg1, Fabp7, Fabp5, Ftl1, Actb, Cpe) are associated with neuronal differentiation and metabolic regulation of the nervous system^33,41^. The first branch trajectory C3$\rightarrow$C8$\rightarrow$C1 corresponds to the Pyramidal cells’ maturation; the second trajectory C3$\rightarrow$C8$\rightarrow$C6 corresponds to the Granule cells’ maturation; the third branch trajectory C3$\rightarrow$C5$\rightarrow$C7 corresponds to the Oligodendrocyte cells’ maturation; the last branch trajectory C3$\rightarrow$C5$\rightarrow$C2$\rightarrow$C4 corresponds to the Astrocyte cells’ maturation. As shown in Fig. 4e, it can be observed that the correlation among genes is undergoing alterations, and diversity indices, as well as cluster coefficients, can be employed to quantify these temporal changes. In Figs. S3f and S3g, the Astrocyte cells maturation trajectory C3$\rightarrow$C5$\rightarrow$C2$\rightarrow$C4 have similar expression patterns in terms of diversity and cluster coefficient metrics, progressively increasing at T= 0, 0.38, 0.64, and 0.87, which may represent Astrocyte cell maturation with multiple cellular activities proceeding. This could prove the applicability of scRDEN for large-scale datasets when identifying the multiple important subgroups, underlying gene-gene expressional networks and dealing with multilineage developing cells.

### Performance comparison

**Fig. 5:**
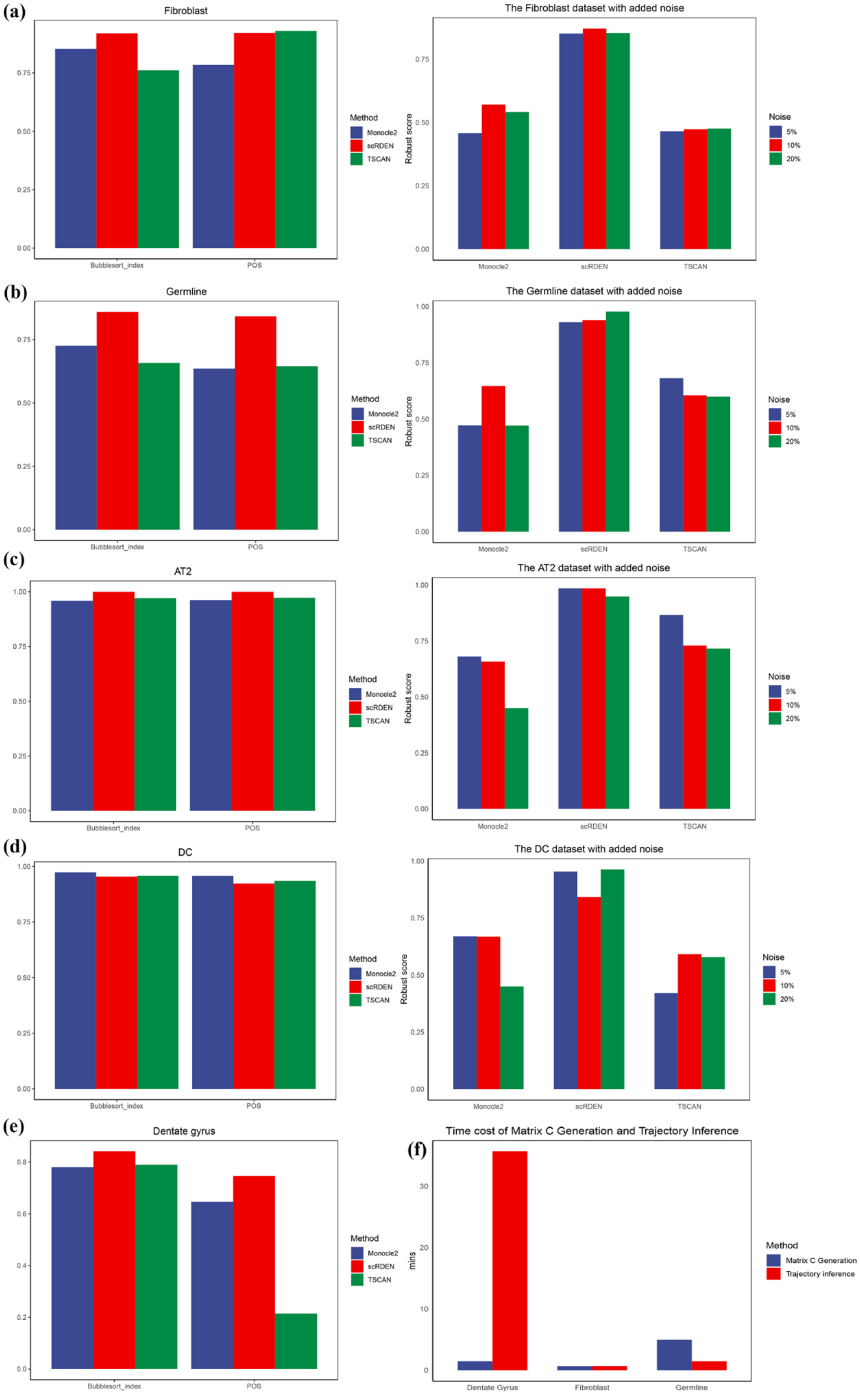
Comparison of the robustness of trajectory inference and comparison of the time complexity of running three datasets. (**a**–**d**) Comparison of the Bubblesort${\_}$index and POS indices for measuring the accuracy of three pseudo-trajectory inference methods (Monocle2, scRDEN and TSCAN) using four datasets under noiseless conditions and with the addition of three types of noise, respectively. The blue color represents the Monocle2 method, the red color represents the scRDEN method, the green color represents the TSCAN method (left). Comparison of the Robust score indices for measuring the stability of three pseudo-trajectory inference methods (Monocle2, scRDEN and TSCAN) using four datasets under the addition of three types of noise (5${\%}$, 10${\%}$, 20${\%}$), respectively. The blue color represents 5${\%}$ noise added to the data, the red color represents 10${\%}$ noise added to the data, the green color represents 20${\%}$ noise added to the data (right). (**e**) The Bubblesort${\_}$index and POS indices for measuring the accuracy of three pseudo-trajectory inference methods (Monocle2, scRDEN and TSCAN) using the large-scale datasets under noiseless conditions. (**f**) The time consumed for Matrix C Generation and Trajectory inference on the three datasets, with the blue color representing the time consumed for Matrix C Generation and the red color representing the time consumed for Trajectory inference.

Since the gene rank differential expression network has been constructed from the gene expression matrix, all subsequent analyses that are typically carried out on the gene expression matrix, such as clustering and trajectory inference, can be performed on scRDEN from the perspective of edges (gene-gene interactions) using any existing scRNA-seq analysis method. Several scRNA-seq analysis methods are further selected to compare the performance of scRDEN with that of other methods in terms of clustering and trajectory inference. Among the trajectory inference methods, TSCAN^23^ and Monocle2^25^have outperformed most of the existing methods, and thus were chosen for comparison with scRDEN in trajectory inference. In the comparative analysis, the Bubblesort${\_}$index^42^ and Pseudo-Temporal Ordering Score (POS) were applied to evaluate the accuracy of the inferred pseudo-time trajectories in Fig. 5. The robustness of scRDEN is also compared with that of other methods by adding 5-20${\%}$ Gaussian noise to the original data. In the comparison of trajectory inference, the Robust score is computed under different levels of noise to quantify the robustness of each proposed trajectory inference method. In the five noiseless datasets^29–33^, the performance of scRDEN outperforms that of the other two classes of trajectory inference algorithms overall. Specifically, it is significantly better than the suboptimal method on AT2, Germline, and Dentate gyrus datasets. In the DC dataset, the POS of scRDEN is more than 90${\%}$, slightly lower than Monocle2(0.96), TSCAN (0.93). However, on all perturbed datasets, scRDEN shows relatively better performance as its Robust score is approximately 20${\%}$ higher than that of the other algorithms. In the AT2 dataset with 20$\%$ Gaussian noise added, the Robust score of scRDEN is approximately 0.95, which is higher than that of the suboptimal method TSCAN (0.71). In the DC dataset with 20${\%}$ Gaussian noise added, the Robust score of scRDEN is 39${\%}$ higher than that of TSCAN. In the Fibroblast dataset with 20${\%}$ Gaussian noise added, the Robust score of scRDEN is about 0.85, approximately 31${\%}$ higher than that of the suboptimal algorithm Monocle2. The three datasets of Dentate gyrus, Fibroblast, and Germline are further utilized to evaluate the running time. In the scRDEN method, this process can be roughly divided into two steps: Matrix C Generation and Trajectory inference, as shown in Fig. 5f. The Dentate gyrus dataset consists of 11,926 cells and 13,999 genes. After screening out the low-expressed genes, the number of genes decreases. Consequently, the step of Matrix C Generation is less time-consuming. In contrast, the Trajectory inference step is more time-consuming due to the large number of cells. However, the overall time consumption is still at a gratifying level (less than 40 minutes). Both the Fibroblast dataset (3379 genes $\times$ 355 cells) and the Germline dataset (5319 genes $\times$ 666 cells) are fast in terms of trajectory inference due to the relatively small number of cells. However, the step of Matrix C Generation is more time-consuming for the Germline dataset compared to the Fibroblast dataset because of the large number of related genes obtained from the Germline dataset. More specific data details can be found in Fig. S5 of the Supplementary Materials. Overall, scRDEN performs well in trajectory inference and clustering compared with other methods.

## Discussion

In this paper, we constructed the gene rank differential expression matrix from gene expression matrices using scRNA-seq data, providing new ideas for trajectory inference and biological significance exploration. To solve the problem of instability and heterogeneity of scRNA-seq gene expression and to better integrate the advantages of reduction methods, we developed scRDEN, which aims to study cell development and differentiation on the relative expression order of genes. We first constructed a gene association matrix by Pearson correlation coefficient and selected a suitable threshold to construct a gene co-expression matrix, from which the associated genes were obtained to convert the unstable gene expression to the difference of relatively stable gene rank. Then three dimensionality reduction methods, PCA, t-SNE, and UMAP, were used to obtain multiple low-dimensional representations, and more accurate clustering results were obtained by taking advantage of the complementary nature of the data, more accurate pseudo-time ordering was obtained by calculating the distance between classes. scRDEN method had better robustness and accuracy than previous trajectory inference algorithms, and the gene-to-gene relationships obtained from gene co-expression networks enable better identification of key genes in the clusters and access to their functions and pathways in cellular dynamic processes. Despite the many advantages of scRDEN method, there are still some drawbacks: one is that the number of clusters found with spectral clustering is relatively small, which may affect the spectral structure of cell differentiation in large datasets, for example, in mouse dentate gyrus dataset, we empirically chose eight classes instead of those determined by spectral clustering. The number of cell clusters in large-scale datasets will be the focus of our next work. Another is that co-expression patterns are also very important for cell fate decisions, and we will apply the ESCO^21^ method to challenge the scRDEN ranking scheme through increasing sparsity of co-expression modules, incorporating the process of co-expression into the study of trajectory inference and regulatory mechanisms.

## Methods

scRDEN method integrates the expression of reliable genes (global features) and rank differential expression of gene pairs (network features) to provide a robust framework for analyzing differences in cell clusters, trajectories, and key regulatory mechanisms in single-cell transcriptional data. Unlike conventional methods that focus solely on gene expression differences, scRDEN selects strongly correlated gene pairs, quantifies their rank differences in cellular expression, and combines the topological relationships of gene expression to characterize cell differentiation dynamics. Moreover, it integrates multiple types of dimensionality-reduced features of cells under the rank-differential expression of gene pairs, not relying solely on a certain type of local feature, and stably infers the multi-branch trajectories and the interaction network of marker genes at each pseudo-time point. Subsequently, combined with the associated gene, the rank differential expression network among genes at each pseudo-time is further inferred. To further investigate and quantify the changes in the gene rank differential expression network at each stage, several indicators of network diversity and clustering coefficients are also used to measure the topological changes of the network with the cell differentiation process. The details of the scRDEN workflow are as follows:

### Preprocessing the gene expression matrix

The dropout events and high noise of the data will affect the real expression of genes in cells. To follow up the reasonable construction of the gene co-expression network, the gene expression matrix is preprocessed in four steps.S1:Preliminary gene screening: to better study the expression of relevant genes in biological development and other processes, mitochondrial genes, spike-in genes, and ribosomal genes are screened out according to gene name, usually ranging in dozens, while retaining genes that are expressed in more than 10$\%$ of the cells. S2: Outlier smoothing: Processing the abnormally expressed genes after the screening of genes, check whether the expression range of each gene is within the range of [$\mu$ -4$\sigma$,$\mu$ +4$\sigma$] by calculating the average expression $\mu$ and variance expression $\sigma$ of each gene in all cells. If the boundary is exceeded, the expression of the gene is smoothed to the gene expression corresponding to the boundary value to obtain the gene expression matrix $A_0$. Valid gene expression in single-cell data can be retained by smoothing the outliers. S3: log transformation: Before selecting the feature genes, in order to eliminate the quantitative differences in gene expression, the gene expression matrix $A_0$ obtained after initial screening and anomaly processing was log2 transformed as follows:

1 $$\begin{aligned} {{A}_{1}}={{\log }_{2}}({{A}_{0}}+1) \end{aligned}$$

where $A_1$ denotes the normalized expression data. In order to maintain the consistency of expression values of 0, when performing log2 transformation, we add a pseudo-count of 1 to the gene expression values. S4: Feature gene selection: To effectively avoid the interference of external factors and capture the stability of the cell population, the intrinsic entropy model developed by Li^43^ was chosen to select feature genes, which is based on the information theory that the intrinsic entropy and extrinsic entropy of a gene actually characterize the intrinsic fluctuation and the ‘noisy’ fluctuation of the gene, respectively. Intrinsic entropy captures the degree of regulatory fluctuation of each gene during cellular processes, and informative genes can be identified from scRNA-seq data to cluster and classify cells accurately. We selected the high intrinsic entropy genes with average expression greater than *I* after log transformation (*I* is a human-selected threshold) to get the high-quality expression matrix $E_{m,n}$ (*m* is the number of genes, *n* is the number of cells) to prepare the processing for the next step of network construction.

### Constructing gene co-expression network

To get the gene co-expression network first need to find the related genes, Chen^28^ directly used the a priori background network which integrates the associated genes from seven databases (including KEGG, Reactome, Biocarta, NCI, SPIKE, HumanCyc and Panther) and got a total of 164,862 pairs of interrelated genes from the databases^44^. However, this prior background network, although supported by multiple databases, is unable to do anything about the genes that do not appear in the databases and may even mask the true gene interactions as a result. In this regard, we consider constructing gene co-expression networks by searching for pairs of genes that may be associated with each other from the gene expression level, which not only greatly reduces the reliance on prior information, but also enables the search for specific associated genes at the single-cell transcriptional level in different organisms. We calculate the linear correlation between genes to get the correlation coefficient matrix based on the Pearson coefficient. Firstly, based on the preprocessed gene expression matrix $E_{m,n}$,the gene correlation coefficient matrix $R_{m,m}$ is obtained by calculation:

2 $$\begin{aligned} {{R}_{m,m}}=\frac{\sum \limits _{i=1}^{m}{({{y}_{i}}-\overline{y})({{z}_{i}}-\overline{z})}}{\sqrt{\sum \limits _{i=1}^{m}{{{({{y}_{i}}-\overline{y})}^{2}}}}\sqrt{\sum \limits _{i=1}^{m}{{{({{z}_{i}}-\overline{z})}^{2}}}}} \end{aligned}$$

${y}_{i}$ and ${z}_{i}$ denote the expression of genes in row *i* and *j* of the gene expression matrix $E_{m,n}$, respectively, and the matrix $R_{m,m}$ represents the Pearson correlation coefficients among *m* genes, with the value ranging from $[-1,1]$, and further correlation test of the matrix $R_{m,m}$ is performed to retain the correlated genes with a significant level (p-value < 0.01). Differently expressed genes will have a more pronounced correlation than other genes^15^, and we do not differentiate between positive and negative correlation effects, and use the magnitude of their absolute value as a measure of their correlation strength property:

3 $$\begin{aligned} {{B}_{0=}}|({{R}_{m,m}})| \end{aligned}$$

where $B_0$ is the absolute-valued matrix. In order to further remove indirect correlations between genes as much as possible based on non-zero correlation coefficients, we use the WGCNA^45^ method to compute a suitable ‘hard’ threshold $b_0$ and retain pairs of genes with correlations greater than the threshold, identifying the following gene co-expression network *B*.

4 $$\begin{aligned} \begin{aligned}&B=G\left( {{V}_{B}},{{E}_{B}} \right) \\&{{e}_{ij}}=\left\{ \begin{matrix} 1 & if\begin{matrix} {{b}_{ij}}>{{b}_{0}},{{b}_{ij}}={{\left( {{B}_{0}} \right) }_{ij}} & \\ \end{matrix} \\ 0 ,& otherwise \\ \end{matrix} \right. \\ \end{aligned} \end{aligned}$$

where ${V}_{B}$, ${E}_{B}$ are the node set and the connected edges set of the network *B*, respectively, ${E}_{B}$ contains the element $E_{ij}$, if the correlation between the *i*th gene and the *j*th gene exceeds the threshold, then $E_{ij}$ = 1, which indicates that there is a direct association between two genes in the co-expression network *B*, and the *i*th and *j*th genes record the node set ${V}_{B}$ of the co-expression network *B*.

### Construction of the gene rank differential expression network

The expression level of genes varies in size between samples, and analyses based directly on gene expression can result in unstable experimental results. Therefore, using a more stable relative expression ordering for analysis can ensure the stability of the results^46^. In this paper, the construction of scRDEN is divided into two steps: S1: Rank matrix ${C}_0$ sorting: the cell expression matrix ${E}_{m,n}$ of the screened genes is transformed into the rank matrix ${C}_0$ according to the size of the expression, and the expression value of each gene in the statistical gene expression matrix is transformed into the rank in cells, and the lower the value of the gene expression, the smaller its rank, and the higher the value of the gene expression, the greater its rank. the calculation criteria of ${C}_0$ are as follows:

5 $$\begin{aligned} \begin{aligned}&{{\left( {{C}_{0}} \right) }_{ik}}=\underset{1\le j\le m}{\mathop {Rank}}\,\left( {{E}_{ij}} \right) \uparrow ,{{\left( {{C}_{0}} \right) }_{ij}}\le m,{{\left( {{C}_{0}} \right) }_{ij}}\in {{Z}^{+}} \\&i=1,2,...n;j,k=1,2,...,m \\ \end{aligned} \end{aligned}$$

Fixed *m*, indicates that the expression value of *i* gene in the *m*th cell is ranked in the *k*th position according to the ascending order, and ${Z}^{+}$ denotes the positive integer. S2: Gene rank differential expression network construction: according to the constructed gene co-expression network *B*, combined with ${V}_{B}$ and ${E}_{B}$, all the associated gene pairs are extracted, and the calculation criteria are as follows:

6 $$\begin{aligned} \begin{aligned}&C=G\left( {{V}_{c}},{{E}_{c}} \right) \\&{{c}_{gt}}={{\left( {{C}_{0}} \right) }_{{{g}_{k}}t}}-{{\left( {{C}_{0}} \right) }_{{{g}_{l}}t}},{{c}_{\text {g}t}}\in {{E}_{c}} \\&{{g}_{k}},{{g}_{l}}\in {{V}_{B}},{{e}_{kl}}=1,{{g}_{k}}\times {{g}_{l}}\in {{V}_{c}} \\&1\le k,l\le n;1\le t\le m \\ \end{aligned} \end{aligned}$$

Where ${V}_{c}$,${E}_{c}$ denote the set of nodes and the set of connected edges of the rank-differentially expressed network *C*, respectively, and ${{g}_{k}}\times {{g}_{l}}$ denote the set of gene pairs consisting of gene *k* and gene *l*, where all gene pairs constitute the set of nodes in ${V}_{c}$. Gene *k* and gene *l* are related in the co-expression network *B*, which is obtained by subtracting the rank of ${{\left( {{C}_{0}} \right) }_{{{g}_{l}}t}}$ from the rank of ${{\left( {{C}_{0}} \right) }_{{{g}_{k}}t}}$ in cell *t*. The rows of the adjacency matrix *c* of the gene rank differential expression network *C* represent the associated gene pairs, and the columns represent the cells. Since the ranks of the genes vary widely, normalization is used for matrix *c* to avoid the effect of extreme values:

7 $$\begin{aligned} c=\frac{{{c}_{i,j}}-\min ({{c}_{j}})}{\max ({{c}_{j}})-\min ({{c}_{j}})} \end{aligned}$$

$\min ({{c}_{j}})$ denotes the minimum value of gene expression in the *j*th column of the adjacency matrix *c*. $\max ({{c}_{j}})$ denotes the maximum value of gene expression in the *j*th column of the adjacency matrix. The normalized gene rank differential expression network matrix is still recorded as *c*. Due to the large size of the gene co-expression network, which is in the hundreds of thousands to millions, the constructed gene rank differential expression network can be cumbersome. We select gene pairs with coefficients of variation in the top 1$\%$ in the adjacency matrix *c* for subsequent computational analysis to reduce the amount of data of a certain size and improve the reasonable use of memory.

### Cell subpopulations and trajectory inference

Compared with the original gene expression matrix, the obtained gene rank differential expression network *C* is sorted according to the size of the expression value, which reduces the influence of the ‘dropout’ events to a certain extent, and measures the more stable differential expression. We used ensemble clustering to obtain more stable clustering results. Firstly, we extracted the features of t-SNE, PCA, and UMAP for matrix *c* and calculated the distance matrix in each dropout space.

8 $$\begin{aligned} {{\rho }_{(v)}}({{x}_{i}},{{x}_{j}})=dist({{x}_{i}},{{x}_{j}}),v=1,2,3 \end{aligned}$$

where ${{\rho }_{(v)}}({{x}_{i}},{{x}_{j}})$denotes the Euclidean distances of cell ${{x}_{i}}$ and cell ${{x}_{j}}$ in the three reduced dimensional spaces. Let the cell similarity network be represented as graph $G=({{V}_{d}},{{E}_{d}})$, vertex ${{V}_{d}}$ corresponds to cell $\{{{x}_{1}},{{x}_{2}},...,{{x}_{n}}\}$, edge ${{E}_{d}}$ represents the similarity of the cells, and the edge weights are represented by $n*n$ similarity matrix *W*, where ${{W}_{i,j}}$ denotes the similarity between cells ${{x}_{i}}$ and ${{x}_{j}}$. Firstly, the average value of the distance between cell ${{x}_{i}}$ and its neighboring cells ${{\rho }_{i\_}}$ is calculated.

9 $$\begin{aligned} {{\rho }_{i\_}}=\frac{\sum \limits _{h=1,...k}{(\rho ({{x}_{i}},{{N}_{h}}))}}{k} \end{aligned}$$

${{N}_{h}}$ denotes the *k* cells with the closest Euclidean distance to cell ${{x}_{i}}$. *k* is an artificially set hyperparameter (default value is 30). Similarly, the average value ${{\rho }_{j\_}}$ of the distances between cell ${{x}_{j}}$ and its neighboring cells is computed, and ${{\varepsilon }_{i,j}}$ is computed to solve the scaling problem:

10 $$\begin{aligned} {{\varepsilon }_{i,j}}=\frac{{{\rho }_{i\_}}+{{\rho }_{j\_}}+\rho ({{x}_{i}},{{x}_{j}})}{3} \end{aligned}$$

The distance matrix is then transformed into a similarity matrix *W*,

11 $$\begin{aligned} {{W}_{i,j}}=\exp (-\frac{{{\rho }_{(v)}}{{({{x}_{i}},{{x}_{j}})}^{2}}}{\mu {{\varepsilon }_{i,j}}}) \end{aligned}$$

where $\mu$ is a hyperparameter set empirically to normalize the similarity matrix *W* to obtain the normalization matrix *P*,

12 $$\begin{aligned} P(i,j)=\left\{ \begin{aligned}&\frac{{{W}_{i,j}}}{2\sum \nolimits _{k\ne i}{{{W}_{i,k}}}},j\ne i \\&\frac{1}{2},j=i \\ \end{aligned} \right. \end{aligned}$$

The sparse kernel *S* is the local affinity matrix between the cell ${{x}_{i}}$ and its neighboring node ${{N}_{i}}$:

13 $$\begin{aligned} S(i,j)=\left\{ \begin{aligned}&\frac{{{W}_{i,j}}}{2\sum \nolimits _{k\ne {{N}_{i}}}{{{W}_{i,k}}}},j\in i \\&0,otherwise \\ \end{aligned} \right. \end{aligned}$$

*P* contains all the information about the similarity of each cell to all other cells, while *S* contains only the information of each cell to the *k* cells with the highest similarity. The algorithm always takes *P* as the initial state and *S* as the kernel matrix in the fusion process, which can capture the local structure of the graph and improve the computational efficiency^47^. According to the dimensionality reduction methods of t-SNE, PCA, and UMAP, we compute the normalization matrices and sparse kernels of the three similar matrices according to equations (12) and (13). These matrices are computed iteratively as follows:

14 $$\begin{aligned} {{P}_{t+1}}^{(v)}={{S}^{(v)}}*(\frac{\sum \nolimits _{k\ne v}{{{P}_{t}}^{(k)}}}{m-1})*{{({{S}^{(v)}})}^{T}},v=1,2,3 \end{aligned}$$

${{P}_{t+1}}^{(v)}$ is the state matrix after *t* iterations of the *v* th matrix, and when the maximum number of iterations is reached or the state matrix is no longer changing, the matrix is no longer updated. The overall similarity matrix *E* is calculated:

15 $$\begin{aligned} E=\frac{1}{v}\sum \limits _{i=1}^{v}{{{P}^{(i)}}} \end{aligned}$$

For the overall similarity matrix *E*, we use spectral clustering to determine the number of clusters and calculate the Laplace matrix $L=D-E$, where *D* denotes the degree matrix. We get the eigenvalue $\lambda$ and eigenvector *x* by $Lx=\lambda x$ , and define the eigenvalue difference ${{eigengap}_{i}}={{\lambda }_{i+1}}-{{\lambda }_{i}}$ , where ${{\lambda }_{i}}$ is the *i*th eigenvalue. Sort ${{eigengap}_{i}}$ according to the size, select the top two eigengap values and select the *i* value of the eigengap of the smaller one as the number of clusters *p* for spectral clustering. Use the clustering results to calculate the pseudo-time ordering, and use the reduced data to infer the trajectories of the cells. The starting clusters ${d}_{1}$ were established based on a priori knowledge, and the centroids of the individual clusters were calculated:

16 $$\begin{aligned} {{d}_{p}}=\frac{1}{{{G}_{p}}}\sum \limits _{i=1}^{{{G}_{p}}}{{{X}_{i}}} \end{aligned}$$

Where ${{G}_{p}}$ denotes the number of cells in the *p*th cluster and ${{X}_{i}}$ denotes the position of each cell in the *p*th cluster in the t-SNE dimensionality reduction space, the distance between the starting cluster ${d}_{1}$ and the other clusters was calculated using the Euclidean distance dist to sort the clusters:

17 $$\begin{aligned} \begin{aligned}&{{D}_{u}}=dist\left( {{d}_{u}},{{d}_{1}} \right) , \\&T_{f}^{v}=\underset{1\le u\le p}{\mathop {Rank}}\,\left( {{D}_{u}} \right) \uparrow ,f\in C{{C}_{v}} \\ \end{aligned} \end{aligned}$$

where ${{D}_{u}}$ denotes the distance between the centroid of the cluster of class *u* and the starting cluster ${d}_{1}$, and $T_{f}^{v}$ denotes the pseudo-time of cell *f* in the cell cluster of class *v* as the position in ascending order among all the center distances. According to the distance between the cluster to which the cell belongs and the starting cluster, the same pseudo-time is assigned to cells in the same cluster.

### Visualizing and analyzing the differences of gene rank differential expression network over pseudo-time.

To further study the changes of gene rank differential network in the process of biological development, cell differentiation and other processes, we construct the network of the dynamics of the rank differential expression network of related genes. After obtaining the pseudo-time of the cell, the dynamic expression of the gene rank differential expression network is determined by selecting a portion of genes and finding out the associated gene pairs constituting the gene rank differential network.

18 $$\begin{aligned} \begin{aligned}&{{H}^{pt}}=G\left( {{V}_{h}},{{E}_{h}} \right) \\&h_{kl}^{pt}=\frac{1}{u}\sum \limits _{w=1,2...u}{\left( h_{{{g}_{k}}w}^{pt}-h_{{{g}_{l}}w}^{pt} \right) },u\in {{G}_{p}},pt\in {{T}_{f}} \\&h_{kl}^{pt}\in {{E}_{h}},{{g}_{k}},{{g}_{l}}\in {{V}_{h}} \\&{{H}_{sym}}^{pt}={{H}^{pt}}+{{({{H}^{pt}})}^{T}} \\ \end{aligned} \end{aligned}$$

where ${V}_{h}$, ${E}_{h}$ denote the set of nodes and the set of edges of the rank differential network of a cell when the assigned pseudo-time is *pt*. *pt* is the pseudo-time assigned to the cell. $h_{kl}^{pt}$ denotes the average of the rank of gene *k* minus the sum of the ranks of gene *l* in the *u* cells assigned a pseudo-time of *pt*. ${{g}_{k}},{{g}_{l}}$ denotes the nodes where gene *k* and gene *l* form the rank differential network. ${{h}^{pt}}_{{{g}_{k}}w},{{h}^{pt}}_{{{g}_{l}}w}$ denotes the rank of gene *k* and gene *l* in the *w*th cell at the assigned pseudo-time *pt*. ${{H}_{sym}}^{pt}$ denotes the rank differential network obtained after symmetrization. In this paper, we mainly focus on the changes in the gene rank differential expression network. The question of the choice of an optimal threshold, although important, will be left open^20^. For better visualization, the edges between genes in the network below the threshold (0.2) will be discarded. By visualizing the rank differential network at different pseudo-times, the most critical periods of biological activity can be identified by observing the topological changes of the network. Entropy has been widely used in statistical mechanics, thermodynamics, and information theory as a measure of disorder or uncertainty in a system^48^. A complex network of molecular interactions describes the cellular phenotype. Elucidating network properties that distinguish the disease from the healthy cellular state is therefore of critical importance for gaining systems-level insights into disease mechanisms^49^. Network entropy could reveal network complexity. Specifically, a network with higher complexity possesses higher network entropy and lower energy and internal energy^50^.Diversity^51^ is similar to the network entropy, which is defined as the scaled Shannon entropy of the weights of its incident edges. Diversity is defined as:

19 $$\begin{aligned} Diversity(i)= & \frac{SE(i)}{\log {{k}_{i}}} \end{aligned}$$

20 $$\begin{aligned}{SE(i)}= & -\sum \limits _{j=1}^{{{k}_{i}}}{{{p}_{ij}}\log }{{p}_{ij}} \end{aligned}$$

where *SE*(*i*) on behalf of Shannon Entropy ,${k}_{i}$ is the degree of node *i*, and ${p}_{ij}$ represents the normalised weights, which is defined by:

21 $$\begin{aligned} {{p}_{ij}}=\frac{{{w}_{ij}}}{\sum \limits _{l\in {{k}_{i}}}{{{w}_{il}}}} \end{aligned}$$

where ${w}_{ij}$ is the weight between gene *i* and gene *j*. Similarly, the cluster coefficient is defined as:

22 $$\begin{aligned} CO(i)=\frac{2TR(i)}{{{k}_{i}}({{k}_{i}}-1)} \end{aligned}$$

*TR*(*i*) is the closed triangle formed by neighboring nodes of node *i*, and ${k}_{i}$ is the degree of node *i*. The cluster coefficient measures how tightly connected a node’s neighbors are. The cluster coefficient and diversity provide a good measure of the topology of a network.

### Evaluation metrics

The ARI and NMI are used to evaluate the accuracy of clustering methods. ARI measures the degree of coincidence between two distributions and ranges from -1 to 1. The closer the value is to 1, the more consistent the clustering result is with the true state. NMI measures the similarity of two clustering results and ranges from 0 to 1. Higher NMI values indicate higher similarity between the two clustering results. To evaluate the accuracy of the trajectory inference algorithms, we utilize the Pseudo-Temporal Ordering Score(POS)^23^, Kendall Correlation, and Bubble Sort Index^42^ to evaluate the accuracy of the inferred trajectory’s cell ordering compared to the true ordering^23^. It is assumed that external information not used in pseudo-time reconstruction is available to evaluate the pairwise order of cells. The POS is calculated as:

23 $$\begin{aligned} POS= & \sum \limits _{i=1}^{N-1}{\sum \limits _{j:j>i}{g(i,j)}} \end{aligned}$$

24 $$\begin{aligned} g(i,j)= & \left\{ \begin{aligned}&0,T(i)=T(j) \\&(T(j)-T(i))/D,otherwise \\ \end{aligned} \right. \end{aligned}$$

where *N* is the number of cells, the *i*th and *j*th cells in the inferred trajectory are collected at time points *T*(*i*) and *T*(*j*), respectively. The variable *D* is chosen to normalize POS so that POS$\in [-1,1]$. Assuming that we know the actual collection time for each cell in the dataset, we can define the position of the inferred pseudo-time ordering as a measure of how closely the order of each pair of cells in the pseudo-time ordering matches the real collection time. If two cells are collected at the same time point, $g(i,j)=0$; otherwise, *g*(*i*, *j*) is positive if *T*(*i*) represents an earlier time point, or negative if *T*(*i*) presents a time point later than *T*(*j*). POS=1 represents that the inferred order of cells perfectly matches the order determined by the cells’ collection time. POS = – 1 means that the inferred order is in the opposite direction of the true situation. A higher absolute POS indicates a more accurate pseudo-time trajectory inference by the algorithm. We assess the robustness of methods by calculating the robust score^23^ between the pseudo-time ordering of the original data and that of the perturbed data. The perturbed data sets are generated by adding Gaussian noise with a scaling factor *k*(*k*=5$\%$, 10$\%$, 20$\%$). The robust score is defined as:

25 $$\begin{aligned} {{W}_{{{o}_{1}},{{o}_{2}}}}=\frac{2}{\left| U \right| (\left| U \right| -1)}\sum \limits _{i,j\in U;i\ne j}{h({{o}_{1}},{{o}_{2}},i,j)} \end{aligned}$$

where ${o}_{1}$ and ${o}_{2}$ denote two pseudo-time orderings respectively, *U* is the union of ${o}_{1}$ and ${o}_{2}$, and $\left| U \right|$ is the cardinality of *U*. If the order of cell *i* and cell *j* is the same in ${o}_{1}$ and ${o}_{2}$, ${h({{o}_{1}},{{o}_{2}},i,j)}=1$, and ${h({{o}_{1}},{{o}_{2}},i,j)}=0$ otherwise. A high robust score indicates that the two cell pseudo-time orderings are similar.

## Supplementary Information

Supplementary Information.

## Data Availability

The AT2, DC and Fibroblast, Germline, Dentate gyrus dataset were obtained from Gene Expression Omnibus (GEO) database under the accession code GSE52583, GSE60783, GSE67310, GSE86146 and GSE104323 respectively.
