# Supplementary material for: scRDEN: single-cell dynamic gene rank differential expression network and robust trajectory inference
Source: Sci Rep. 2025 May 15;15:16963. doi: 10.1038/s41598-025-01969-1 (PMC12081924; doi:10.1038/s41598-025-01969-1)
Supplement: Supplementary file 1 — Supplementary Information. [file 41598_2025_1969_MOESM1_ESM.docx]

**scRDEN: Single-cell dynamic gene rank differential expression network and robust trajectory inference**

**Han Zhang^1^, Wei Zhang ^1^, Xiaoying Zheng ^1*^, and Yuanyuan Li ^1*^**

**Supplementary Materials**

**
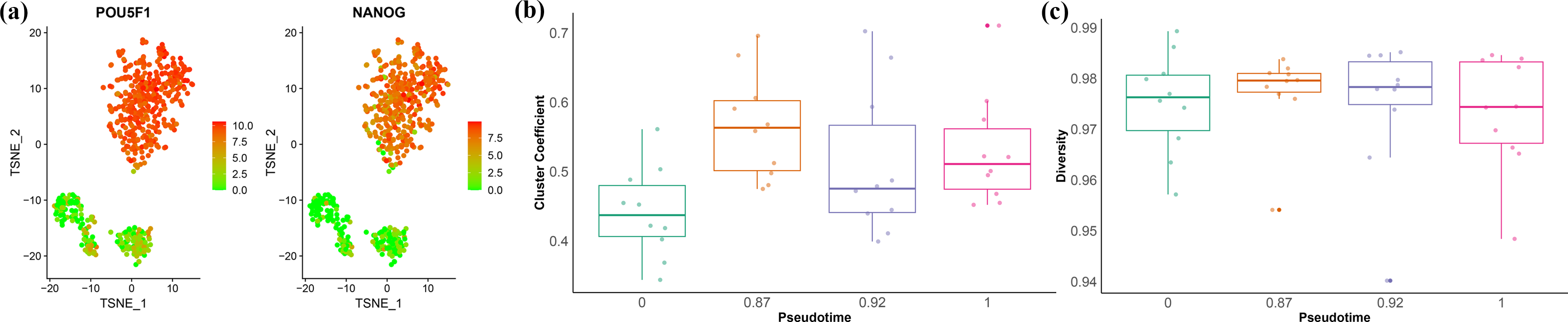
**

Figure S1. (a) Heatmap of expression of the marker gene POU5F1 and NANOG for the initial time point of germline dataset.(b) Performance of cluster coefficients for four networks over pseudo-time.(c) Performance of diversity for four networks over pseudo-time.


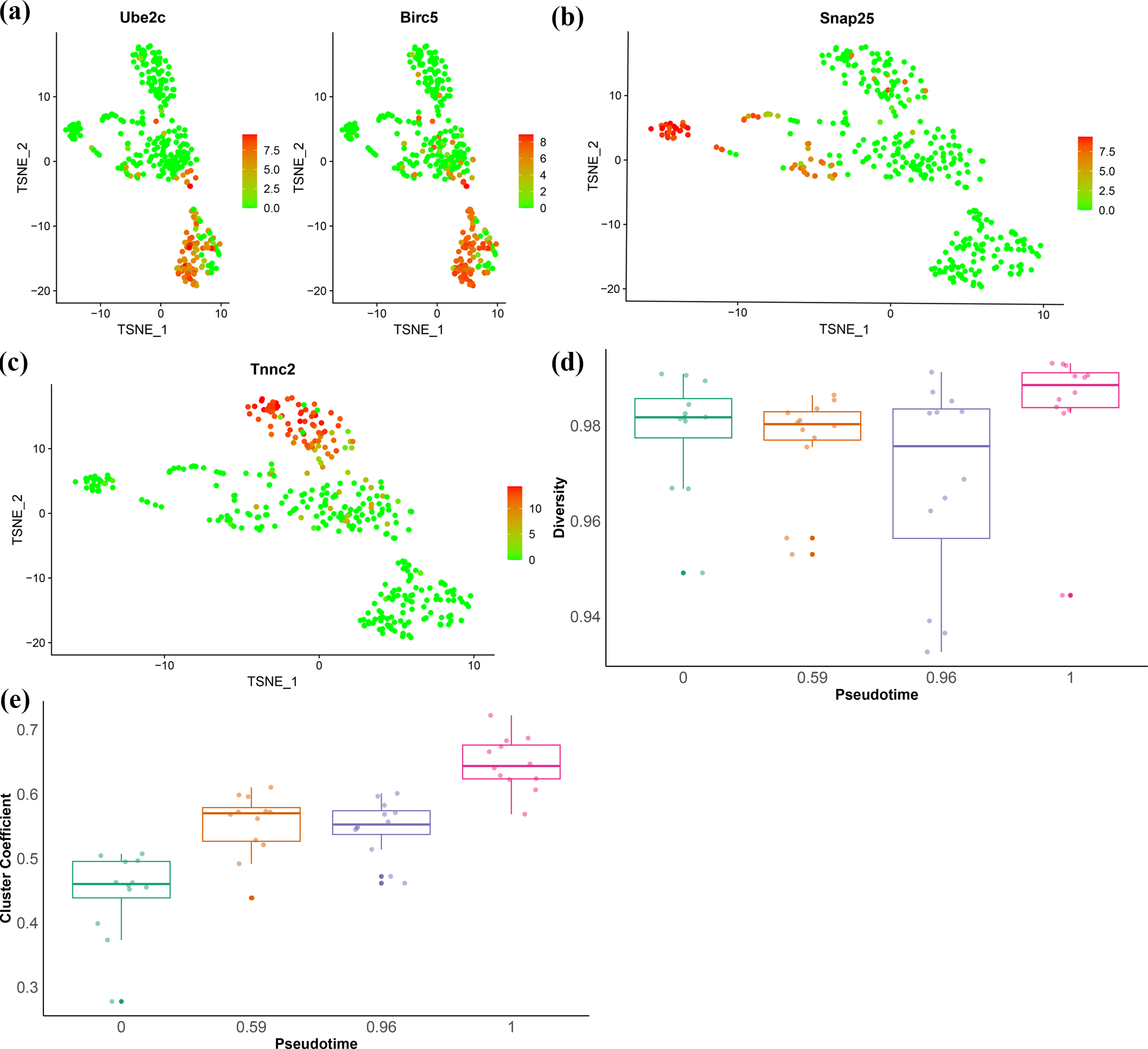


Figure S2. (a) Heatmap of expression of the marker gene Ube2c and Birc5 for the initial time point of mouse embryonic fibroblasts dataset. (b) Heatmap of expression of the marker gene Snap25 in Neuron cells.(c) Heatmap of expression of the marker gene Tnnc2 in Myocyte cells.(d) Performance of diversity for four networks over pseudo-time. (e) Performance of cluster coefficients for four networks over pseudo-time.


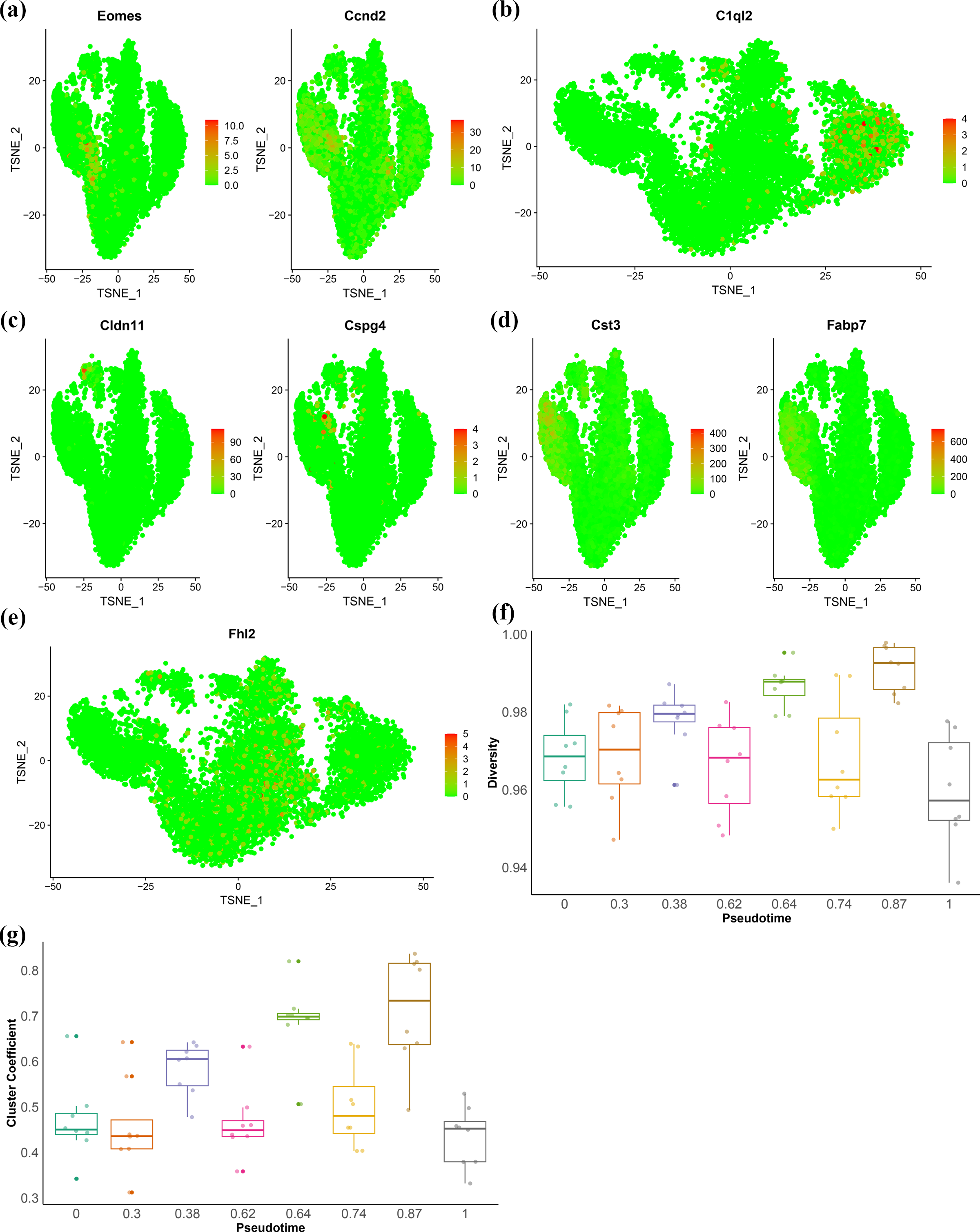


Figure S3. (a) Heatmap of expression of the marker gene Eomes and Ccnd2 for the initial time point of mouse dentate gyrus dataset.(b) Heatmap of expression of the marker gene C1ql2 in Granule cells.(c) Heatmap of expression of the marker gene Cldn11,Cspg4 in Oligodendrocyte cells. (d) Heatmap of expression of the marker gene Cst3,Fabp7 in Astrocyte cells. (e) Heatmap of expression of the marker gene Fhl2 in Pyramidal cells. (f) Performance of diversity for eight networks over pseudo-time. (g) Performance of cluster coefficients for eight networks over pseudo-time.
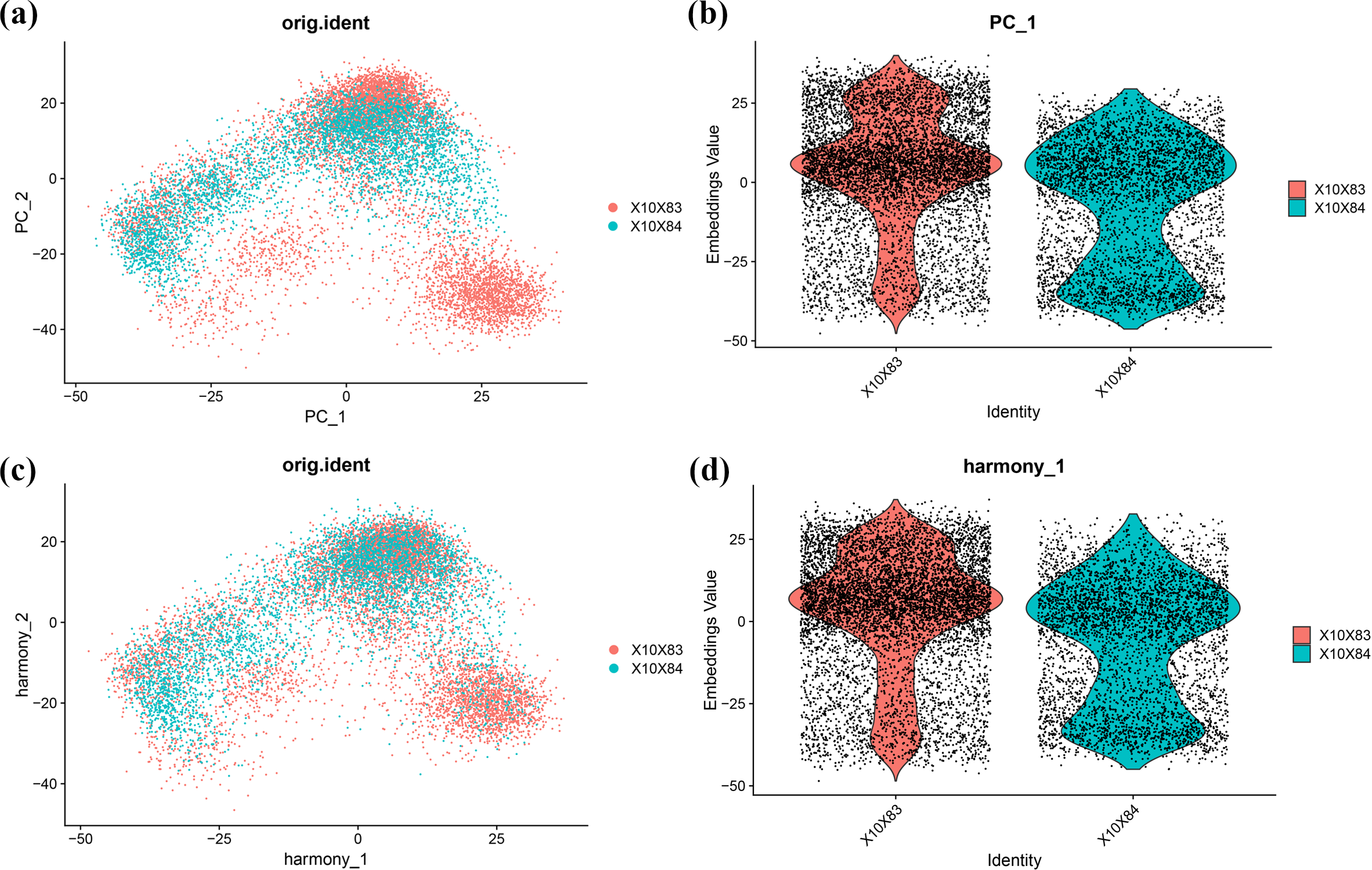
Figure S4. Dimensionality reduction plots of cells and distribution of embedding values, different colors represent different batches. (a)The PCA dimensionality reduction plot without removing the batch effects. (b)Distribution of embedding values of the cells without removing the batch effects. (c)The Harmony dimensionality reduction plot after removing the batch effects. (d) Distribution of embedding values of the cells after removing the batch effects.


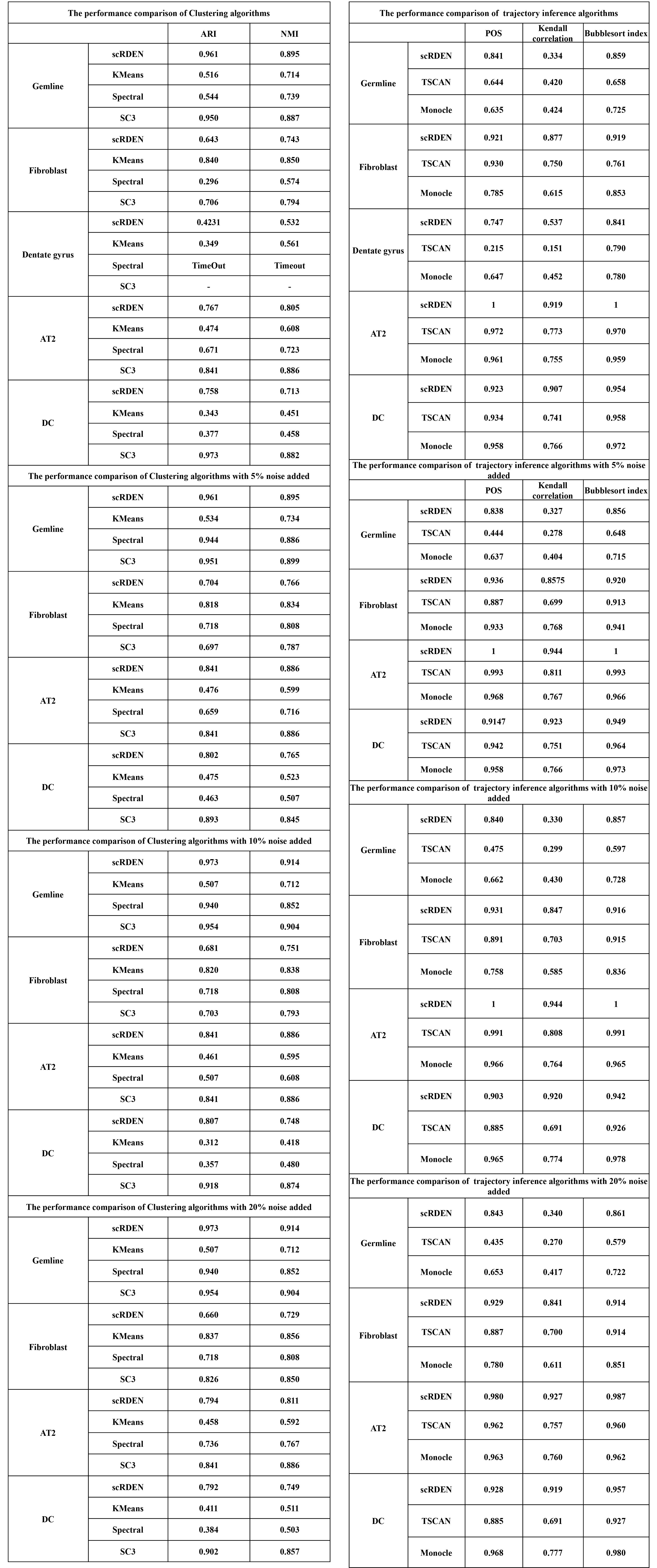


Figure S5. Metrics for clustering and trajectory inference for raw and noise-added dataset.
